# Effect of Dopaminergic Medication on Postural Sway in Advanced Parkinson’s Disease

**DOI:** 10.3389/fneur.2013.00202

**Published:** 2013-12-13

**Authors:** Fredy J. Revilla, Travis R. Larsh, Ashutosh Mani, Andrew P. Duker, Cyndy Cox, Paul Succop, Maureen Gartner, Claudia Jarrin Tejada, Amit Bhattacharya

**Affiliations:** ^1^Department of Neurology, University of Cincinnati College of Medicine, Cincinnati, OH, USA; ^2^Gardner Center for Parkinson’s and Movement Disorders, University of Cincinnati Neuroscience Institute, Cincinnati, OH, USA; ^3^Veterans Affairs Medical Center, Cincinnati, OH, USA; ^4^Biomechanics-Ergonomics Research Laboratory, Department of Environmental Health, University of Cincinnati College of Medicine, Cincinnati, OH, USA; ^5^Department of Environmental Health, Division of Epidemiology and Biostatistics, University of Cincinnati College of Medicine, Cincinnati, OH, USA; ^6^Department of Internal Medicine, Virginia Commonwealth University Medical Center, Richmond, VA, USA

**Keywords:** Parkinson’s disease, postural, balance, sway, dyskinesia, fall, dopaminergic, levodopa

## Abstract

**Background:** The effect of dopaminergic therapy on balance in Parkinson’s disease (PD) remains unclear, including previous studies that excluded the effect of dyskinesias or other involuntary movements on postural sway. Additionally, medication’s effects may differ between fallers and non-fallers. In this study, the authors quantify the effect of dopaminergic medication on postural balance (sway) in advanced PD, with and without dyskinesias, and consider the patient’s history of falls.

**Methods:** In 24 patients with advanced idiopathic PD, postural balance was measured using a strain-gage force platform. Before and after taking dopaminergic medication, the patient’s postural sway was measured at 30-s intervals to determine sway length (SL) and sway area (SA). Data analysis included the presence of dyskinesias during “ON” medication condition and history of previous falls.

**Results:** No significant changes occurred in SL or SA with dopaminergic treatment for fallers without dyskinesias or non-fallers with dyskinesias. However, after dopaminergic treatment, SL and SA were 37.8 and 45% lower, respectively, in non-fallers without dyskinesias (indicating better balance) and were 87.4 and 162.8% higher, respectively, in fallers with dyskinesias (indicating poorer balance). In the ON-medication condition, SL and SA were larger in patients with dyskinesias when compared with patients without dyskinesias; SL was larger in fallers than non-fallers in both groups with or without dyskinesias.

**Conclusion:** Dopaminergic medication effects on postural sway could be a predictive factor for fall risk in PD patients with and without dyskinesias: specifically, decreased sway could indicate minimal fall risk whereas no change or increased postural sway could indicate a high risk.

## Introduction

Factors contributing to balance impairment in Parkinson’s disease (PD) patients include disturbed postural reflexes, poor control of voluntary movements, and side effects of certain medications that include dyskinesias and orthostatic hypotension (1, 2). The effect of dopaminergic therapy on postural balance remains controversial. Counterintuitively, several studies have found that postural sway, a marker of balance impairment characterized by motion of the body’s center of pressure (CoP), is increased by dopaminergic treatment in patients with advanced PD (3, 4). However, these studies did not account for the effects of dyskinesias or other involuntary movements on postural sway. Nova et al. concluded that dopaminergic medications improved subjective measures of postural stability in PD patients when measured using the Berg functional balance scale and the motor subscale of the Unified Parkinson’s Disease Rating Scale (UPDRS-III) (5). Nevertheless, doubts remain about the reliability of subjective measures of postural stability, such as the pull test of the UPDRS-III (6). In another study of patients in the early stages of the disease, Beuter et al. demonstrated significant improvement in objective measures of postural sway after acute dopaminergic treatment (7). However, balance is typically preserved in the first few years of PD and dyskinesias are uncommon (8).

With effects of dopaminergic medications on postural balance examined by others, we uniquely quantify the effect of dopaminergic medication on postural balance (sway) in 24 patients with advanced PD, with and without dyskinesias, and consider the patient’s history of falls. Given the importance of identifying predictors of fall risk and objectively assessing the effect of treatment options on postural balance, we examined sway length (SL) and area in patients with and without dyskinesias, and with and without a history of falls.

## Materials and Methods

Recruited by the University of Cincinnati (UC) Movement Disorders Center, our 24 patients had idiopathic PD [according to standard criteria (9)] (Table 1). During the recruitment period, patients with advanced PD and motor fluctuations, some of which who were being considered as potential candidates for deep brain stimulation (DBS), were offered participation in this study.

**Table 1:** **Patient demographics in 24 patients with advanced PD classified with or without dyskinesia and as faller or non-faller**.

Demographic		No dyskinesia group (*n* = 11)		Dyskinesia group (*n* = 13)	
		Non-fallers (*n* = 7)	Fallers (*n* = 4)	Non-fallers (*n* = 4)	Fallers (*n* = 9)
Sex		Five male, two female	Three male, one female	Four male	Five male, four female
**FINDING (MEAN ± STANDARD ERROR)**					
Age years		63.9 ± 3.7	62.7 ± 4.2	58.0 ± 2.2	60.9 ± 2.6
Disease duration years		13.4 ± 2.0	8.0 ± 1.8	11.0 ± 0.9	10.2 ± 1.4
Levodopa equivalent dose (mg)		614.3 ± 65.9	565.5 ± 78.2	716.0 ± 171.1	513.4 ± 66.0
UPDRS-III rating	OFF	36.0 ± 1.8	40.0 ± 1.6	38.9 ± 5.4	30.3 ± 2.3
	ON	17.2 ± 2.8	18.9 ± 5.5	17.5 ± 1.5	15.2 ± 2.0
Axial UPDRS rating	OFF	7.1 ± 0.7	8.3 ± 0.6	9.0 ± 0.6	8.1 ± 0.5
	ON	4.0 ± 0.8	4.5 ± 1.2	4.3 ± 1.1	3.6 ± 0.4
Postural stability rating	OFF	0.7 ± 0.3	0.8 ± 0.5	1.3 ± 0.5	0.7 ± 0.2
	ON	0	0.3 ± 0.25	0	0

Patients underwent an evaluation in the “practical off” state (after overnight fast of at least 12 h from dopaminergic medications) and after dopaminergic treatment. For transition to on-state, patients were given their usual dose of medications, except for a few who took a higher dose than usual. Two patients who take amantadine for dyskinesia as part of their daily medication regimen were not given amantadine on the day of testing. The time between taking dopaminergic medications and on-state testing averaged 1 h 50 min. All patients received and gave informed consent with protocols approved by the UC Institutional Review Board. Patients did not receive monetary compensation for their participation.

### Clinical evaluation

The UPDRS-III was used for each medication condition (10). The axial subscore was derived from UPDRS items 18, 22 (neck rigidity only), 27, 28, 29, and 30.

### Objective measure of balance

Postural balance or sway was measured using a strain-gage type force plate platform system (Model #OR6-5-1000, Advanced Mechanical Technology, Inc.) as previously described (11). Parameters of static posturography were collected for 30 s before and after dopaminergic treatment (OFF- and ON-medication conditions, respectively) (11, 12). Patients were instructed to stand upright, relax their arms at their sides, and look straight ahead during testing. They wore a safety harness as a precautionary measure to prevent falls during testing. Four trials were done in each state.

### Data analysis

During each test condition, the force platform system provided six channels of signals, that is, three orthogonal forces (*F*_x_, *F*_y_, *F*_z_) and three moments around three orthogonal axes (*M*_x_, *M*_y_, *M*_z_); these signals were processed off-line with our custom made software (Posture60 and KineLysis, University of Cincinnati, 2000). The force platform system was calibrated as previously described (11). Briefly, the customized software calculates *x*-*y* coordinates of the patient’s CoP during testing (11, 12) and then plots the movement relative to this center during a trial (stabilogram) that included a variety of related outcome measures. Most often, two measures are used: (1) sway area (SA) describing the total area encompassed by the stabilogram (in the *x*-*y* or the horizontal plane) during each test period; and (2) SL describing the total length of movement relative to CoP during each test. Subsequently, an increase in SA, SL, or both implies worse postural stability.

To compare the effect of dyskinesias on postural sway measurements, patients were classified based on the presence or absence of dyskinesias. During the ON-medication condition, using the Modified Dyskinesia Scale, we divided the 24 patients based on the presence or absence of dyskinesia: with dyskinesia (*n* = 13) for rating ≥1 or without dyskinesia (*n* = 11) for rating zero; both were for a minimum of 9/12 trials. All ratings were performed by a Movement Disorders neurologist (Fredy J. Revilla or Andrew P. Duker). The Modified Dyskinesia Scale rates the severity of whole-body dyskinesias on a scale of 0–4 (where a rating of 4 represents highest severity of dyskinesia) and has excellent inter- and intra-rater reliability (13).

Any history of falls was obtained from patient report and scrutiny of medical records from within 2 years before evaluation. History of falls was treated as a binary variable; patients with at least one fall were classified as *fallers* and patients without falls were classified as *non-fallers*. Groups, regardless of fall history or presence of dyskinesias, did not differ significantly in age, sex, or disease duration. Levodopa equivalent doses (LEDs) were comparable between the groups (Table 1).

### Statistical analysis

For statistical analysis, SL and SA were transformed to their natural logarithms to ensure normal distribution. ANOVA (repeated measures) was used to assess significance. Significant interactions were followed up with *post hoc* tests (Tukey’s HSD tests). An alpha level of 0.05 was used for all tests. Statistical analysis was performed using R version 2.10.1.

## Results

In quantifying effects of dopaminergic medication on postural balance measures of SL and SA, we found no significant differences among our 24 patients with or without dyskinesias and with or without a history of falls with respect to clinical measures (UPDRS-III, axial subscore, and postural stability) in either medication ON or OFF condition (Table 1). However, clinical measures (UPDRS-III, axial subscore, and postural stability) improved, regardless of fall history and presence of dyskinesias during testing in the ON state.

Mean (±SEM) LED from all PD medications for the dyskinesia and no dyskinesia groups were 576 (±70) and 597 (±49), respectively. Nine out of 13 PD patients with dyskinesia and 7 out of 11 PD patients without dyskinesia took dopamine agonist medications. Mean (±SEM) LED from dopamine agonists for the dyskinesia and no dyskinesia groups were 186 (±55) and 189 (±46), respectively. Hence, the dyskinesia and no dyskinesia groups were not significantly different from each other with respect to medications.

**Figure 1:**
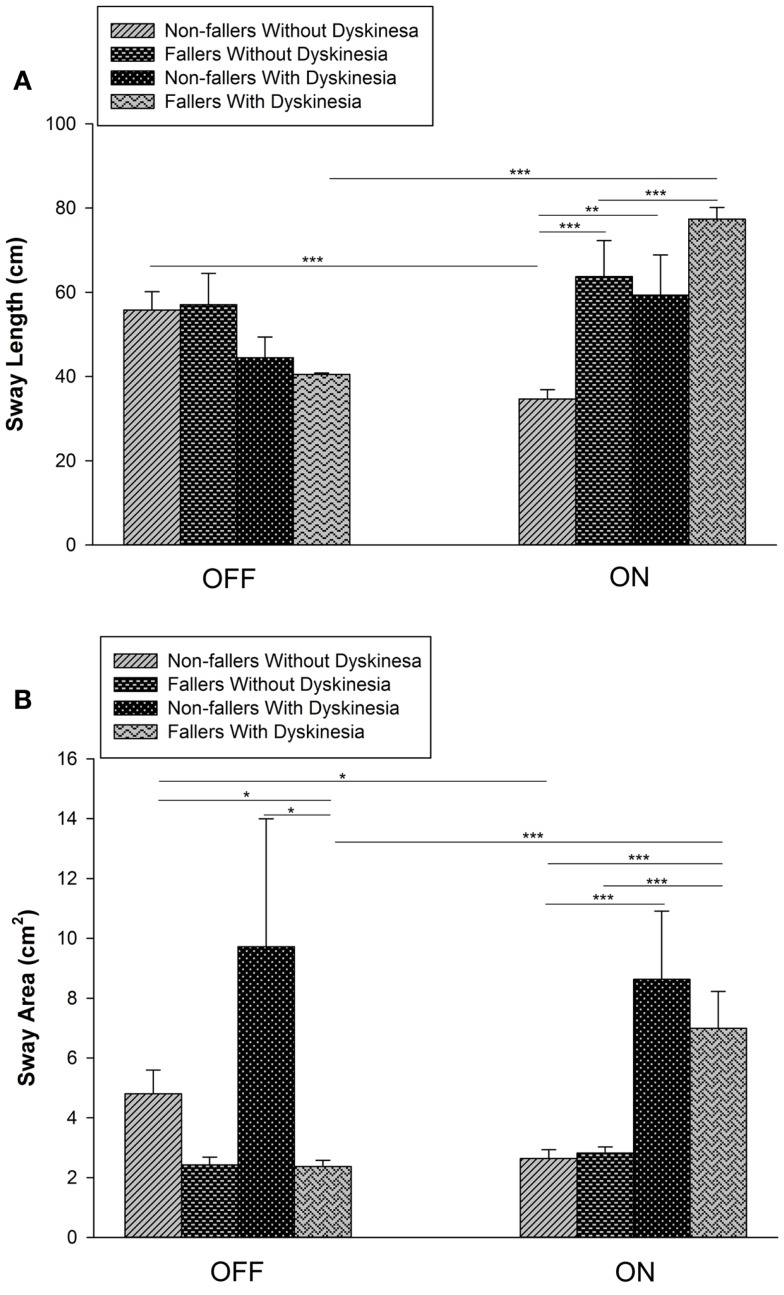
**Effect of dopaminergic medication on postural sway length (SL) and area (SA)**. **(A)** Sway length decreased significantly in non-fallers without dyskinesia but increased significantly in fallers with dyskinesia. In the ON state, SL was significantly greater in fallers than non-fallers without dyskinesia. **(B)** Sway area increased significantly in fallers with dyskinesia and decreased significantly in non-fallers without dyskinesia. In the ON state, SA was significantly greater in both fallers and non-fallers with dyskinesia than those without dyskinesia (*p < 0.05; **p < 0.01; ***p < 0.001).

In the ON-medication condition, non-fallers without dyskinesias improved in both objective and subjective measures of balance testing, demonstrating improved postural balance with acute dopaminergic treatment (Figure 1; Table 1). Specifically, for ON medication, both SL and SA were larger in patients with dyskinesias compared with patients without dyskinesias; SL was larger in fallers than non-fallers with or without dyskinesias (*p* < 0.001).

In the OFF medication condition, mean SL did not differ significantly between groups with or without dyskinesias (Figure 1). However, SA was lower in fallers with dyskinesias than in non-fallers with (*p* = 0.004) or without (*p* = 0.001) dyskinesias.

No significant changes occurred in SL or SA with dopaminergic treatment for fallers without dyskinesias or non-fallers with dyskinesias. However, in the ON-medication condition, SL and SA were 37.8% (*p* < 0.001) and 45% (*p* = 0.02) lower, respectively, in non-fallers without dyskinesias (indicating better balance) and were 87.4% (*p* < 0.001) and 162.8% (*p* < 0.001) higher, respectively, in fallers with dyskinesias (indicating poorer balance) (Figure 1).

Mean dyskinesia scores (mean ± SEM) for fallers and non-fallers were 1.42 ± 0.14 and 1.75 ± 0.23, respectively. None of the PD patients had a dyskinesia rating of 4. Using ANOVA by treating dyskinesia scores as factors, dyskinesia scores were found to be highly significant for SL (*p* < 0.001) and SA (*p* < 0.001).

## Discussion

In our 24 patients with advanced PD, we found association between differential effect of dopaminergic medication on postural balance and fall history. In groups with or without dyskinesia, we could discriminate fallers from non-fallers based on SL values. Although SL in the ON-medication state was greater in fallers than non-fallers (Figure 1), further study is needed to determine if postural sway measurements can become a tool to identify which non-faller patient may be at risk for falls. Further research should investigate the effects of dopaminergic medication; specifically, does decreased postural sway indicate minimal fall risk and no change or increased postural sway indicate a higher risk. For example, the magnitude of SL in the ON-medication state may be useful to identify which patients are at risk of falls. In the ON-medication state, non-dyskinetic non-faller PD patients had SL comparable with that of healthy adults (of comparable age). Poor balance in PD can be implied given the relatively higher measurements in SL and area in our study groups in both medication states than that found in healthy adults ages 60–69 years (SA: 2.2 ± 1.38 cm^2^ and SL: 36.37 ± 10.11 cm for healthy adults).

Although effects of dopaminergic medications on postural balance have been examined in other studies (Table 2), our study uniquely analyzed postural sway data of patients based on dyskinesias and fall history. In the ON-medication condition, non-fallers without dyskinesias improved in both objective and subjective measures of balance testing, demonstrating improved postural balance with acute dopaminergic treatment (Figure 1; Table 1). However, dopaminergic treatment did not significantly alter sway parameters for non-fallers with dyskinesias (Figure 1); this discrepancy is presumably due to the effect of dyskinesias on CoP. Specifically, any extraneous movement causes a shift in CoP location. In addition, we found that dyskinesia score had a significant effect on both SL and SA, meaning that postural sway was affected by dyskinesia severity. Therefore, the presence of dyskinesias in these patients possibly masked improvements in postural balance that resulted after dopaminergic treatment (14).

**Table 2:** **Summary of studies exploring the effect of dopaminergic medication on postural balance and shortcomings of each study**.

Reference	Major finding	Study limitation
Rocchi et al. (3)	Dopaminergic medication	Did not explore relationship between postural sway, history of falls, and dyskinesias
	Increased root mean square of CoP distance from the center of sway and SA in six patients with advanced PD	
	Increased sway more in the mediolateral than in the anterior-posterior direction	
	Interestingly decreased mean sway velocity	
Contin et al. (4)	Dopaminergic medication	Did not explore relationship between postural sway, history of falls, and dyskinesias
	Increased SL and SA in 32 patients with early PD and 24 patients with advanced PD	
Armond et al. (15)	Dyskinesias increased postural sway parameters	Included only dyskinetic patients
	Severity of dyskinesias correlated with level of postural sway	Did not explore relationship between postural sway and history of falls
Beuter et al. (7)	Dopaminergic treatment decreased postural sway in patients with early stages of PD	Included only patients with early PD
		Did not explore relationship between postural sway, history of falls, and dyskinesias
Nova et al. (5)	Dopaminergic treatment improved postural balance, as evaluated by Berg’s functional balance scale	Used strictly subjective measures
		Did not explore relationship between postural sway, history of falls, and dyskinesias
Revilla et al.	In the absence of dyskinesias, dopaminergic medication	Binary faller/non-faller with/without dyskinesia classifications
	Decreased SL and SA in non-fallers	
	Did not significantly affect SL or SA in fallers	
	In the presence of dyskinesias, dopaminergic medication	
	Did not significantly affect SL or SA in non-fallers	
	Increased SL and SA in fallers	

### Postural sway factors and balance

The observed disconnect between the degree of postural sway (measured by force platform) and UPDRS-III probably reflects the fact that this particular measurement of postural reflexes does not account for the effect of dyskinesias. This interpretation agrees with the findings of others who suggest that dyskinesias may increase the risk of falls (1, 2). The pull test of the UPDRS-III evaluates patients only in their ability to respond to a uniaxial perturbation. Patients with dyskinesias demonstrated an improvement in all aspects of the UPDRS-III, including the pull test. Although this improvement is presumably indicative of the patient’s improved ability to respond to a posteriorly directed perturbation, it cannot comprehensively predict the patient’s ability to maintain their center of gravity (CoG) within their base of support. In a study that described this limitation of the pull test, Bloem et al. found that the test was not adequately predictive of falls (16). An alternative interpretation of our findings may be that CoP measurements are not predictive of postural stability in PD patients with dyskinesias. Rather CoP measurements assess balance based on the assumption that they reflect CoG position (17). Balance control describes the maintenance of the body’s CoG within its base of support whereas postural instability occurs when the CoG approaches near proximity of this base (18). However, CoP measurements are not always indicative of CoG position, especially during non-static conditions (17). Therefore, the presence of dyskinesias introduced a dynamic component that may have influenced the measured CoP. The improvement of subjective measures observed with dopaminergic treatment, even in the presence of dyskinesias, could suggest that patients were better able to maintain their CoG within their base of support.

### Study limitations

Among several limitations, the classification of a patient to be with or without dyskinesia was based solely on this movement disorder during testing. A patient who often has dyskinesia with dopaminergic treatment possibly did not develop dyskinesias during testing; the opposite is also true. The potential bias of our classification as fallers or non-fallers was based on patient’s self-reporting of falls declared during the clinical visit. Given our limited sample size of 24 patients and that one patient’s data could greatly influence results, a larger sample size is needed to further determine the effects of dyskinesias, medication, and fall history on postural balance. Also, because of our limited sample size, we cannot rule out that differences could exist in comparisons where we did not find statistically significant differences. With the sole use of CoP measurements, we could not distinguish between patients who had normal postural control superimposed with dyskinesias versus patients who had abnormal postural sway in addition to dyskinesias (19).

In addition to medication effects, further investigations on how other treatments (e.g., DBS) might influence postural balance and how fallers and non-fallers respond are needed. Relationships between CoG and CoP in PD patients also merit future study.

## Conclusion

Dopaminergic medication appears to affect postural sway among patients with advanced PD based on the history of falls and presence of dyskinesias. Response of postural balance to dopaminergic medication may be predictive of increased fall risk. Dopaminergic medication reduced postural sway for patients with lower fall risk but conversely had negligible or detrimental effects on postural sway for those with a higher fall risk. Owing to our limited sample size, further study on the effect of dyskinesias on postural stability and differences in postural sway between fallers and non-fallers are needed, and may help in determining the optimal doses of dopaminergic medications that provide the maximal possible benefit with the least side effects.

## Author Contributions

Fredy J. Revilla, Travis R. Larsh, Ashutosh Mani, Andrew P. Duker, Cyndy Cox, Paul Succop, Maureen Gartner, Claudia Jarrin Tejada, and Amit Bhattacharya contributed to the review and draft of this paper, and all also approve this final version.

## Conflict of Interest Statement

Fredy J. Revilla, Travis R. Larsh, Ashutosh Mani, Andrew P. Duker, Cyndy Cox, Paul Succop, Maureen Gartner, Claudia Jarrin Tejada, Amit Bhattacharya declare that they have no competing interests. Dr. Fredy J. Revilla is a consultant for Lundbeck and Medtronic.
